# Automated 3D thorax model generation using handheld video-footage

**DOI:** 10.1007/s11548-022-02593-4

**Published:** 2022-03-31

**Authors:** Nadine Dussel, Reinhard Fuchs, Andreas W. Reske, Thomas Neumuth

**Affiliations:** 1 Center of Information Technology and Medical Engineering, University Hospital of Heidelberg, Im Neuenheimer Feld 130.1/130.3, Heidelberg, 69117 Baden-Württemberg Germany; 2 Innovation Center Computer Assisted Surgery (ICCAS), Leipzig University, Semmelweisstr. 14, Leipzig, 04103 Saxony Germany; 3 Department of Anesthesiology, Intensive Care, Emergency Medicine and Pain Therapy, Heinrich-Braun-Klinikum, Karl-Keil-Straße 35, Zwickau, 08060 Saxony Germany

**Keywords:** Image analysis, Photogrammetry, Automated model generation, Emergency medicine, Marker detection

## Abstract

**Purpose:**

For the visualization of pulmonary ventilation with Electrical Impedance Tomography (EIT) most devices use standard reconstruction models, featuring common thorax dimensions and predetermined electrode locations. Any discrepancies between the available model and the patient in terms of body shape and electrode position lead to incorrectly displayed impedance distributions. This work addresses that problem by presenting and evaluating a method for 3D model generation of the thorax and any affixed electrodes based on handheld video-footage.

**Methods:**

Therefore, a process was developed, providing users with the ability to capture a patient's chest and the attached electrodes via smartphone. Once data is collected, extracted images are used to generate a 3D model with a structure from motion approach and locate electrodes with ArUco markers. For the evaluation of the developed method, multiple tests were performed in laboratory environments, which were compared with manually created reference models and differences quantified based on mean distance, standard deviation, and maximum distance.

**Results:**

The implemented workflow allows for automated model reconstruction based on videos or selected images captured with a handheld device. It generates sparse point clouds from which a surface mesh is reconstructed and returns relative coordinates of any identified ArUco marker. The average value for the mean distance error of two model generations was 5.4 mm while the mean standard deviation was 6.0 mm. The average runtime of twelve reconstructions was 5:17 min, with a minimal runtime of 3:22 min and a maximal runtime of 7:29 min.

**Conclusion:**

The presented methods and results show that model reconstruction of a patient’s thorax and applied electrodes at an emergency site is feasible with already available devices. This is a first step toward the automated generation of patient-specific reconstruction models for Electrical Impedance Tomography based on images recorded with handheld devices.

## Introduction

The correct assessment, diagnosis, and pulmonary function monitoring remain a challenge, especially in the prehospital or emergency setting. There, injuries to the chest (thorax) represent one of the most common types of injury [1]. Thoracic trauma is in conjunction with pneumothorax (the accumulation of air in the pleural space) the second leading cause of death in polytrauma patients in Germany. The assessment of lung ventilation by emergency personnel usually starts by auscultation of the lungs with a stethoscope. Due to this procedure's susceptibility to interference from ambient noise, it is of limited use in high-noise scenarios. In most cases, the emergency physician can only suspect the existence and size of a pneumothorax.

Even if there remains some uncertainty about the presence of a pneumothorax, chest tubes are placed as a precautionary measure to remove the potentially threatening accumulation of air from the pleural space. However, studies have shown that 24% of chest tubes placed by emergency physicians would not have been necessary, and up to 22% of drains were not put in the correct position, requiring correction in the hospital [2]. Half of the drains inserted were ineffective and did not resolve the patient's potentially life-threatening pneumothorax. Due to these circumstances, there is a demand for mobile systems that can measure or visualize pulmonary ventilation quickly and in any given scenario.

Recent publications highlight the use of ultrasound in emergencies, where auscultation is difficult due to the environmental noise [3, 4]. Another approach is to use an Electrical Impedance Tomography (EIT) system. EIT gives a more conclusive analysis of pulmonary ventilation. It covers a larger area within the patient's thorax and is more user-friendly as the sensors are placed on the skin and do not need to be held by the user. EIT is a non-invasive, radiation-free imaging technique that can provide information about the lungs' ventilation and function with low-resolution reconstruction images [5, 6]. For these reasons, efforts have been undertaken in recent years to develop handheld devices that enable emergency medical personnel to perform EIT in ambulatory emergencies. The procedure involves placing electrodes around the patient's chest to measure the conductivity of the inside of the body by using weak electrical currents. Measurements taken at different times are compared in a reconstruction algorithm to visualize the intra-thoracic impedance changes subsequently.

The reconstruction of EIT images is an inverse problem since measured values are used to infer the change in their cause [7]. The application of a reconstruction matrix, created and optimized using a 2D or 3D model of the human thorax, solves the inverse problem and determines the changed impedances [8]. With the models' help, the electric current's behavior in the patient's body is approximated so that the shape, extent, and proportioning of the upper body region in the model have a crucial role in the subsequent reconstruction of EIT images [9]. Also, the electrodes' positions in the model take on a defining part, as their location on the upper body and the distances between them significantly affect the approximation of impedance changes.

Therefore, to determine the magnitude and position of intra-thoracic impedance changes with sufficient accuracy, it is necessary to enter the affected patient's anatomy and the location of the attached sensors into the reconstruction model as a priori information. Depending on the individual patient, using a standard model that has deviating characteristics may result in the misrepresentation of impedance changes. If a patients suffers from pneumothorax, a potentially life-threatening air collection between the lung and the inside of the chest-wall, distorted EIT images (reconstructed using standard models not representing the individual anatomy) could identify the location of the pneumothorax incorrectly. Pneumothorax compromising heart or lung function needs to be treated by thoracotomy. The minimum information required would be which side of the chest is affected [10]. Minor impedance shifts in the EIT reconstructions and thus the resulting localization of the pneumothorax have no direct influence on the placement of the drainage, as long as it detects the correct side of the chest. In communication with medical experts we determined that a too large deviation of the model, e.g., a shift of the electrode positions by one place, which corresponds to 30 –40 mm for a belt with 32 electrodes, corrupts the position information too much and any information about the pulmonary ventilation occurring unilateral or bilateral, and in the latter case delayed or symmetric, would be displayed incorrectly.

Hence, the model must be trained or adjusted to be patient-specific, yet manual modeling is not desirable. According to medical experts, a seamless integration of the patient-specific EIT-analysis in a fast-paced emergency medical scenario is only possible if it requires less than 2 min for setup and initial computation, in some cases less than 1 min. Therefore, performing extensive upper body measurements and manual model design with dedicated hardware and software consumes too much time. Instead of initiating time-consuming designing activities, the feasibility of which depends on robust and possibly unwieldy hardware, the modeling or model fitting should be done automatically without the necessity for additional user inputs while employing already available hardware.

## Related work

Based on these requirements, methods for location-independent surface recognition and electrode identification, and electrode localization had to be found. A similar problem exists in studies concerning electroencephalography (EEG). The estimation of spatial coordinates of applied electrodes to the head is essential to minimize source displacement of electrical brain activity. This challenge was addressed by implementing a method for photogrammetric coordinate measurements and color-based identification of EEG electrodes positions on the human head, employing a rotating, 2 MP digital camera in an experimental setup [11]. In a stationary laboratory setup, the approach was used for the identification and localization of electrodes with a maximum error of 0.77 mm.

The present work further develops this approach to determine the positions of electrodes attached to the thorax in an emergency with simple means. For this purpose, 3D models of the patient's thorax with attached electrodes were created using smartphones.

## Materials and methods

The developed method to detect the electrodes and create the 3D model of the patient's thorax consists of two phases, visualized in Fig. 2. The first phase is the data acquisition with a smartphone camera (Phase A: 1), and the second phase is the calculation of the 3D model with the attached electrodes (Phase B: 2–10). To validate this method, the quality of the surface scan and the quality of the electrode positions were determined (Phase C).

### Data acquisition

Before generating the data for the 3D model and the electrode positions, each electrode was prepared with a marker. Without the markers, the pipeline couldn't detect the electrodes. A smartphone camera was used for the data acquisition, allowing emergency medical personnel to record a video of the upper patient's body with already present hardware. Video recording is supposed to be done from one side to the other with an arc-shaped movement and at a steady distance between the patient and the smartphone, as depicted in the first step of Fig. 2.

#### Preparation of the electrodes

**Fig. 1:**
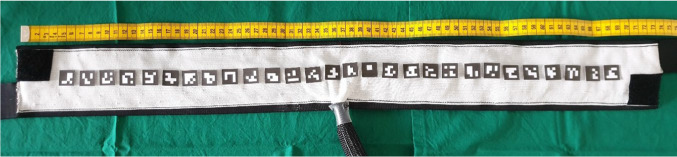
EIT belt with ArUco markers on electrodes and measuring tape

For the belt preparation, 32 Ag/AgCl-electrodes were mounted on a stretchable, textile belt that has a length of 70 cm when it is not stretched. The electrodes were squared and 10 × 10 mm in dimension, however since they are not visible if the belt is worn by the patient, electrode positions were highlighted by *ArUco* markers (Fig. 1). Each *ArUco* marker represented one electrode with its respective index. *ArUco* markers were generated with the *OpenCV* library, using the fastest predefined dictionary; *4* × *4_50*. For the squared markers a surface area of 17 × 17 mm was chosen, which was limited by the distance of the subjacent electrodes toward each other. For *ArUco* marker detection the functions provided by the *OpenCV* library were used as well.

#### Video recordings

Video recordings were performed in a laboratory environment, where the stretchable belt was applied around the thorax of a volunteer as well as two different phantoms. During video capture, the person or object can be standing, sitting upright, laying in an inclined or a supine position. Videos were recorded with a smartphone, which was moved during recording from one side of the target body wearing the EIT belt to the other side in an arc shape with a constant distance of 70 cm between smartphone and body surface. The covered angle of the arc had a radius of 100 degrees, while the movement was performed with a constant speed. For later references, the captured body or phantom surface was measured and the relative electrode positions were recorded alongside a measuring tape.

### Calculation of the 3D model with the attached electrodes

The calculation of the 3D model and the electrode positions is based on images, which were extracted from the video records with two frames per second. The images were used for the generation of sparse point clouds of the captured surfaces, using a structure from motion approach, which was developed by Shilkrot et al. [12].

#### Create the 3D-Model

**Fig. 2:**
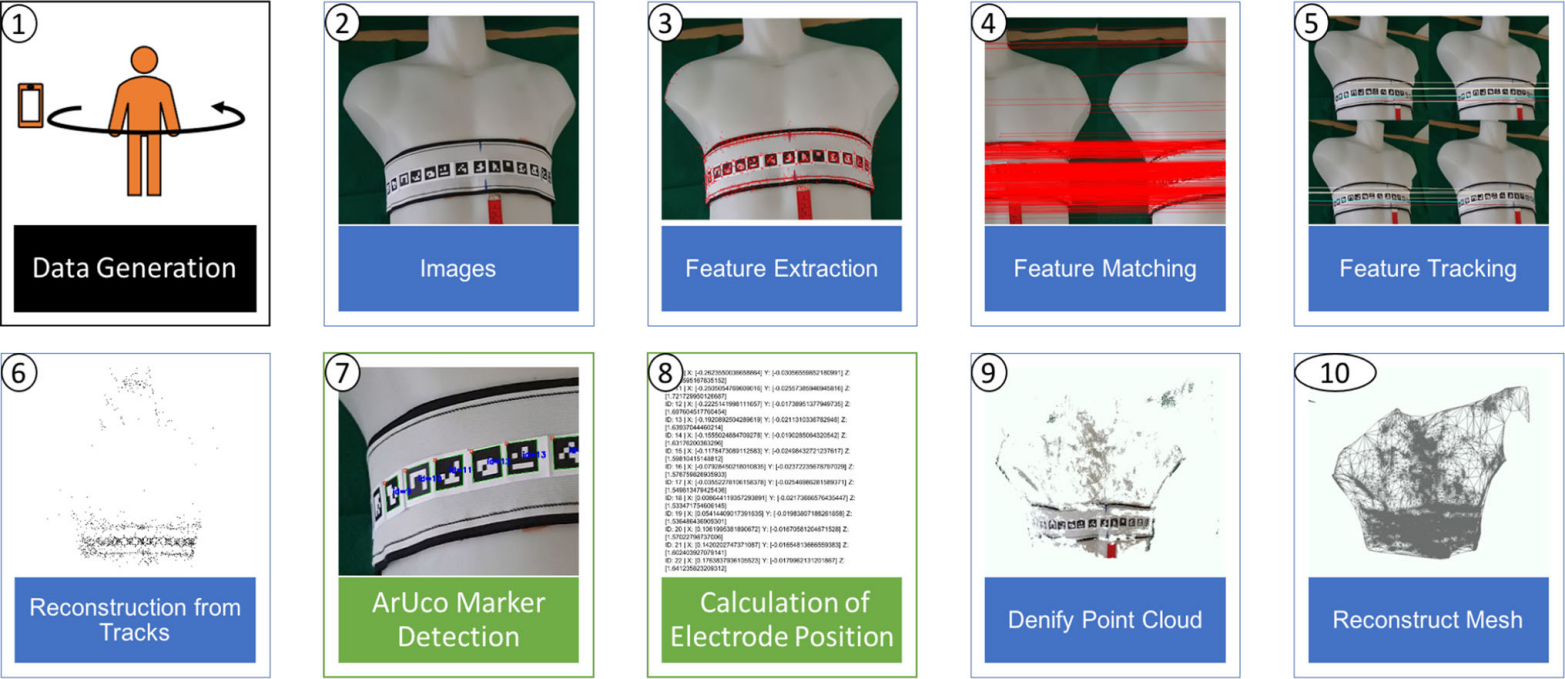
Representation of surface reconstruction pipeline of Shilkrot et al. [11] with one torso model dataset

Shilkrot et al. [12] describe the used pipeline in chapter 2 *Explore Structure from Motion with the SfM Module 2* of the book *Mastering OpenCV 4*. The image processing is visualized in Fig. 2 (blue boxes), which shows how to reconstruct a mesh from multiple images of the desired body surface. After image recording, features in each image were extracted (3) by the AKAZE detector [13, 14]. The detected features were used to define the shared features between two images (4), the possible matches were filtered by multiple methods. Afterward image features were tracked (5) in multiple images by match graphs. Through a reconstruction of the tracking, a sparse point cloud was generated (6) by the structure from motion method [13]. The sparse point cloud was densified (9), using the *OpenMVS* library [15]. The mesh, which represents the surface of the target body area, was created from the resulting dense point cloud (10).

#### Detection of the attached electrodes

The electrode positions were calculated using the *ArUco* markers, which were attached on the opposite side of the belt, facing away from the patient. The markers were detected in each extracted frame of the video recording, thereby marking their corner points (Fig. 2, 7). Found corner points of each marker were defined as a two-dimensional vector within the extracted images. Following the identification, the corner-point-vectors were adjusted, ensuring there existed the same number of markers and identical marker indices. The two positions of one corner point in two adjacent frames were triangulated using the Direct Linear Transformation method (DLT method). Afterward filtering of the triangulated *ArUco* markers was employed to generate four unique three-dimensional vertices per marker and remove multiple occurring vertices, which resulted from the pairwise triangulation of neighboring images. The calculation of the electrodes' middle positions was done by calculating the three-dimensional center point from the extracted corner points of every detected marker. This way the corners of the *ArUco* markers were calculated and the two-dimensional marker points triangulated and filtered (Fig. 2, 8). Subsequently, each detected electrode was defined by the four corner positions in three-dimensional space.

### Method validation

For validation, the video-reconstruction error and the runtime of the method were determined by testing the method on two objects and one person. The error of the video reconstruction was divided into the 3D model reconstruction and the electrode position detection. Each part of the video reconstruction was validated using the open-source software *Cloud Compare*. The standard deviation, the mean difference, and the maximum difference from the reconstructed point clouds to the original point clouds or the electrode positions were measured. Two objects were compared with 12 3D model reconstructions in total. The 3D model reconstructions of two objects and one person were compared with 19 electrode position reconstructions.

#### Error calculation for reconstructed 3D-Model

The reconstructed 3D-Model was scaled manually, registration between the reconstructed model and the original mesh was done by aligning the two models per custom-picked equal point pairs. Fine registration of the reconstructed 3D-Model and the original mesh was performed after aligning the respective entities. Once alignment and fine registration were completed, the distance between mesh and model was calculated.

#### Error calculation for reconstructed electrode-positions

The error calculation for the electrode-positions was done by measuring the 3D-Model and mesh distance and identifying the scale factor between the reconstructions and the body of the volunteer or phantoms. Based on the recorded measurements, which were performed during the video recordings for the model reconstruction, the scale was determined, and identified marker positions and their respective corner locations were scaled to match with the reconstructed model. Afterward, the distances between the reconstructed and the real electrode positions were calculated, by using the distances between adjacent electrodes during the recording to determine their actual positions on the body and the coordinates of their corners.

#### Runtime validation

The method's runtime was measured from twelve reconstructions of an object and seven reconstructions of a phantom. The duration of each step of the pipeline is documented, and the standard deviation is calculated. This is achieved by measuring the runtime duration of each pipeline component in milliseconds, i.e., measuring the amount of time necessary to reconstruct the sparse point cloud, locate markers and assign electrodes, densify the point cloud, and reconstruct the mesh from the dense point cloud. Afterward, the entire runtime of the complete pipeline process is calculated, after which the calculation of the standard deviation of the measured reconstructions is performed.

## Results

**Fig. 3:**
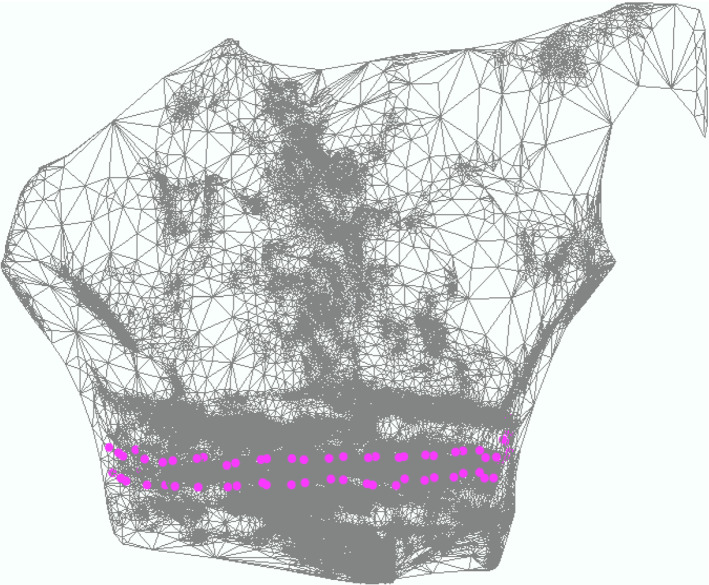
Generated mesh based on measurements with phantom as target object and electrode belt. Mesh is shown in three dimensional view; pink dots mark the corners of fiducial markers over electrodes. The cornerpoints are enlarged and some of them are invisible because of the overlapping mesh

One result of the explained surface scan with a smartphone is shown in Fig. 3. The scanned object, a phantom of the upper body, is presented by a triangular mesh. The pink dots represent the fiducial marker corners which highlight the electrode corners.

### Video reconstruction error

The error distances of the 3D model reconstruction to the original mesh over two objects and twelve reconstructions are shown in Table 1. It has a mean distance of 5.4 mm and a standard deviation of 6.0 mm. The calculated reconstruction errors of the electrode positions are shown in Table 2 for the torso phantom and the second object. Overall, electrode position reconstructions over two objects and twelve reconstructions have a mean distance of 3.7 mm and a standard deviation of 2.5 mm. Counterpart error calculations for the human volunteer are shown in Table 3. The mean distance of electrode position reconstruction error over one person and seven reconstructions were *m* = 7.0 mm with a standard deviation of 6.8 mm.

**Table 1:** Error of video reconstruction on the torso model and the second model

	Mean distance (mm)	Std. dev. (mm)	Max. distance (mm)
Reconstruction T-Model 1	9.80	22.80	219.00
Reconstruction T-Model 2	10.90	20.20	144.40
Reconstruction T-Model 3	6.10	10.80	95.90
Reconstruction T-Model 4	10.30	15.70	123.40
Reconstruction T-Model 5	2.70	10.90	107.40
Reconstruction T-Model 6	2.50	4.20	149.40
Reconstruction T-Model 7	2.80	5.10	62.00
Reconstruction T-Model 8	3.60	8.80	77.70
Reconstruction T-Model 9	3.60	5.80	83.10
Reconstruction S-Object 1	9.30	7.90	49.80
Reconstruction S-Object 2	− 2.00	7.00	42.40
Reconstruction S-Object 3	− 0.90	8.10	40.70
Ø Reconstructions of torso model and second object	5.40	6.00	99.60

**Table 2:** Error of electrode position reconstruction on the torso model and the second object in millimeters

	Mean distance (mm)	Std. dev. (mm)	Max. distance (mm)
Reconstruction T-Model 1	4.40	4.70	21.00
Reconstruction T-Model 2	2.90	2.50	10.30
Reconstruction T-Model 3	7.20	11.00	57.40
Reconstruction T-Model 4	2.50	2.00	8.30
Reconstruction T-Model 5	3.50	3.00	18.10
Reconstruction T-Model 6	2.50	2.30	10.30
Reconstruction T-Model 7	2.30	2.80	17.90
Reconstruction T-Model 8	1.40	1.00	5.60
Reconstruction T-Model 9	3.90	3.90	22.20
Reconstruction S-Object 1	4.30	3.70	13.50
Reconstruction S-Object 2	3.30	2.40	8.50
Reconstruction S-Object 3	6.30	4.70	17.10
Ø Reconstructions of torso model and second object	3.70	2.50	17.50

**Table 3:** Error of electrode position reconstruction on the person in millimeters

	Mean distance (mm)	Std. dev. (mm)	Max distance (mm)
Reconstruction 1	7.30	8.00	30.30
Reconstruction 2	5.80	4.10	18.90
Reconstruction 3	8.20	6.80	24.20
Reconstruction 4	6.60	7.30	29.40
Reconstruction 5	5.10	4.60	20.90
Reconstruction 6	7.30	9.10	47.10
Reconstruction 7	− 6.60	7.40	17.00
Ø Reconstructions	7.00	6.80	26.80

### Runtime

Table 4 presents an overview of the measured runtimes of surface reconstruction with electrode localization on the used torso phantom. The method's total runtime (Phase B) measured on one object with twelve reconstructions has a mean of 5:17 min and a standard deviation of 1:26 min. The minimal measured total runtime is 3:22 min and the maximal 7:29 min. The standard deviation of the total runtime and the twelve reconstructions is 1:26 min. To reconstruct a sparse point cloud, the method needs an average of 0:25 min. The sparse point cloud's densification requires an average computation time of 4:44 min, the following reconstruction of the mesh has a mean runtime of 0:06 min. The detection of the electrode positions requires an average time of 0:02 min. Measured runtimes of surface reconstruction with electrode localization on the volunteering person are shown in Table 5. The total runtime of the method (Phase B) measured on one person with seven reconstructions has a mean of 8:30 min. The standard deviation is 2:39 min. The densification counts on average 07:37 min and has a standard deviation of 2:14 min.

**Table 4:** Runtime of the surface reconstruction with electrode position detection on the torso model and second object

	Sparse point cloud (hh:mm:ss)	Densify point cloud (hh:mm:ss)	Mesh (hh:mm:ss)	Electrode position (hh:mm:ss)	Total (hh:mm:ss)
T-Modell 1	00:00:15	00:03:00	00:00:06	00:00:01	00:03:22
T-Modell 2	00:00:17	00:04:31	00:00:05	00:00:02	00:04:54
T-Modell 3	00:00:36	00:05:47	00:00:01	00:00:03	00:06:28
T-Modell 4	00:00:21	00:04:36	00:00:01	00:00:02	00:05:00
T-Modell 5	00:00:16	00:03:41	00:00:04	00:00:01	00:04:03
T-Modell 6	00:00:28	00:04:13	00:00:07	00:00:02	00:04:50
T-Modell 7	00:00:19	00:03:17	00:00:06	00:00:02	00:03:43
T-Modell 8	00:00:20	00:06:52	00:00:08	00:00:03	00:07:23
T-Modell 9	00:00:24	00:04:33	00:00:09	00:00:02	00:05:08
T-Modell 10	00:00:20	00:03:43	00:00:11	00:00:02	00:04:16
T-Modell 11	00:00:33	00:06:05	00:00:13	00:00:02	00:06:53
T-Modell 12	00:00:46	00:06:35	00:00:05	00:00:03	00:07:29
Mean runt	00:00:25	00:04:44	00:00:06	00:00:02	00:05:17
Std. dev	00:00:09	00:01:18	00:00:04	00:00:00	00:01:26

**Table 5:** Runtime of the surface reconstruction with electrode position detection on the person

	Sparse point cloud (hh:mm:ss)	Densify point cloud (hh:mm:ss)	Mesh (hh:mm:ss)	Electrode position (hh:mm:ss)	Total (hh:mm:ss)
Modell 1	00:00:30	00:05:47	00:00:05	00:00:01	00:06:22
Modell 2	00:00:36	00:07:00	00:00:05	00:00:01	00:07:41
Modell 3	00:00:48	00:07:43	00:00:02	00:00:02	00:08:36
Modell 4	00:00:28	00:07:10	00:00:11	00:00:01	00:07:49
Modell 5	00:01:25	00:11:33	00:00:05	00:00:04	00:13:07
Modell 6	00:00:52	00:09:17	00:00:28	00:00:03	00:10:40
Modell 7	00:00:20	00:04:49	00:00:05	00:00:02	00:05:15
Mean runt	00:00:43	00:07:37	00:00:09	00:00:02	00:08:30
Std. dev	00:00:22	00:02:14	00:00:09	00:00:01	00:02:39

## Discussion

### Video reconstruction error

In regard to the requirements stated in the introduction, which stem from the application of the user-specific EIT-reconstruction for detection and localization of a pneumothorax, the average deviations of the generated models and the calculated spatial positions of the fiducial markers were within a desired range and therefore acceptable. While the largest maximal discrepancies occurred during the reconstruction of the torso model, reaching a mean maximal error distance of 118 mm, the average error distance was 5.8 mm, which means maximal errors of that dimensions are most likely outliers. On average, the error distance in this instance is not even quarter of the 30 mm, the model-reality-electrode-displacement which would be likely to cause major shifts in the impedance distributions. Therefore, the approach is regarded as suitable, once improvements have been made in the post-processing of the method. The model reconstruction of the second model yields similar results with an average mean error distance of 4 mm, while the mean maximal distance is 44.3 mm, far less than the torso model and probably due to a small number of outliers and generation artefacts. The error distances resulting from the spatial localization of the fiducial markers and the connected electrodes are smaller than the model deviations. The average mean electrode displacement errors for the torso model, the ultrasonic phantom and the tests on a volunteer being 3.4, 4.6 and 7 mm, respectively. The average maximal distances are 19 mm for the torso model, 13.1 mm for the ultrasonic phantom and 26.8 mm for the volunteer. Spatial electrode localization based on fiducial marker detection is therefore more accurate with smaller error distances, with the average mean error distances reaching values smaller than 1 cm, supporting the validity of the implemented method.

While clinical evaluation and tests regarding the accuracy increase in EIT with the patient-specific model have yet to be done, the results can already be compared with studies concerning exact electrode placement during electrocardiogram (ECG) measurements. Kelly McCann et al. showed that out of 924 ECG-electrode arrangements, emergency department clinicians reached a mean placement error of 13.5 mm in a vertical and 16.5 mm in a horizontal direction, both errors being higher than mean errors achieved with the presented method [16]. However, Barber and Brown recognized in their studies early on, that uncertain electrode positioning with even small deviations affect the reconstruction outcome and lead to significant EIT image errors [17]. The effect of incorrect electrode locations has been studied further, proving them to be the cause of severe imaging artifacts [18, 19]. With the additional model inaccuracies, caused by movement of the thorax through breathing and postural changes, these image errors increase significantly and ultimately, make EIT images unsuitable for medical diagnosis altogether [20, 21]. Yet many EIT systems rely on the correct placement of a particular electrode belt, whose application doesn't usually result in an electrode pattern with equidistant spacing, increasing differences between 3D models and real-world conditions considerably. Compared to this, the results of the proposed method are an improvement and justify the pursuit of further development steps in that direction.

### Runtime and usability

The presented work is therefore a first step toward patient-specific EIT reconstruction models, yet it needs further work for actual applicability. The processing times during this study were rather long and did not meet the requirements for application in emergency medicine, posed by the medical experts. While data processing is done automatically and requires no additional input after the initial video recording, the shortest runtime of model generation workflow was 3:22 min, extending the maximal processing time of 2 min by over 1 min. The largest processing time frames exceeded 10 min, which is not acceptable in any emergency setting, considering the necessity to evaluate the patients’ ventilation as soon as possible to reduce possibly irreversible damage. Additional measures have to be taken in order to minimize the necessary computing times, especially the point cloud densification, which is currently the biggest time-related bottleneck of the method.

In comparison with previous publications, like Baysal and Şengül, the method presented in this work has a higher usability and is more flexible while being more applicable in pre-clinical situations, since our work requires only a smartphone or tablet and allows small distance variations [11]. However, as a trade-off for the significant reduction in use constraints, the measurement errors of our method are higher. Another flexible approach was presented by Fritsch, employing a smartphone or tablet to capture images to calculate the three-dimensional model of a recorded scene [22]. Contrary to this work, the model creation required an initial calibration of the smartphone/tablet camera, resulting in an unavoidable delay.

### Future works

In order to improve accuracy additional steps for image processing need to be implemented, enabling the medical application of the method by compensating blur, shaky camera movement, and artifacts caused by rain, debris, or scratches on the lens surface. The electrode displacement needs to be addressed in future research, with additional correction steps during the video processing and the post-processing of the mesh generation, minimizing any errors occurring during modeling or spatial detection of fiducial markers. Further development should address inaccuracies and outliers of the model generation with surface smoothing algorithms as well as constraints for neighboring surface elements during post-processing. The effectiveness of edge detection, threshold algorithms, and filter methods need to be evaluated, comparing the cost–benefit ratio of accuracy and processing time.

For the improvement of the model calculation time, the image processing workflows needs to be executed on one or several external, powerful computing machines, which should lead to considerable decreases in the required time to process images and create the adjusted model. To that end, an automated and encrypted end-to-end communication needs to be implemented, which is used with a dedicated handheld application that is not reliant on any stored data, assuring compliance with data protection regulations. This would enable users to transfer encrypted recordings to the external system and receive anonymized model information. The necessary computations could possibly be reduced further, when the implemented method no longer generates a complete model from scratch, but rather uses the image features to adjust an already existing model and place the detected fiducial markers onto it. The approach could include constraints during the matching process, through which neighboring surface elements with unrealistic distances toward each other can be avoided, avoiding outliers. However, model adjustment is a feature based optimization process and could lead to increased inaccuracies as a trade-off for the faster computation or even require more time to present a finished model, if the error gradient thresholds are too demanding. Hence, the first approach should include the comparison of model adjustment results with structure models generated by the methods presented in this paper, ensuring equal or improved accuracy. Finally, the next iteration of the method needs to be evaluated in a dedicated study, analyzing the model generation and electrode-placement error over multiple subjects, preferably with a corresponding 3D model and analyzing its advantages.

## Conclusion

For using EIT in an emergency, a method was developed to detect the surface of a patient's thorax and the electrode positions of the EIT belt by a smartphone video. The created thorax model with the electrodes can be used to select the best patient-specific thorax model for EIT reconstruction. Now, the method's results have to be integrated into the selection of the patient-specific models within the EIT process.

## Data Availability

The datasets (reconstructed 3D models and videos) generated during and analyzed during the current study are available from the corresponding author on reasonable request.

## Code availability

The entire code of the study is saved in a GitLab project of the ICCAS. The code of the 3D reconstruction with the structure from motion approach is available in the Github repository, https://github.com/PacktPublishing/Mastering-OpenCV-4-Third-Edition/blob/master/Chapter_02.
